# Pediatric Unilateral Chronic Sinusitis: Clinical and Radiological Patterns Compared to Adults

**DOI:** 10.22038/IJORL.2024.75788.3539

**Published:** 2024-05

**Authors:** Ahmed Adel Sadek, Maisara Mohamed Mahmoud Nasr, Ahmed Ali Mohamed Badawi, Tamer El Zaeem Esmaeel, Osama Galal Awad

**Affiliations:** 1 *Department of Otorhinolaryngology, Faculty of Medicine, Minia University, Egypt.*; 2 *Department of Diagnostic Radiology, Faculty of Medicine, Minia University, Egypt.*

**Keywords:** Chronic sinusitis, Computed tomography, Unilateral sinusitis, Pediatrics

## Abstract

**Introduction:**

Upper respiratory tract infections and sinusitis are more prevalent in children than in adults. Unilateral sinusitis is not uncommon disease. Our aim was to analyze the disease characteristics in children with unilateral sinusitis and compare them with those of adults.

**Materials and Methods:**

This study included 124 patients with unilateral chronic sinusitis divided according to age into two groups: pediatric group ≤18 years (66 cases) and adult group >18 years (58 cases). The groups were compared in terms of demographic data, side, clinical manifestations and radiological findings.

**Results:**

In pediatric patients, the most common inflammatory pathology was antrochoanal polyps, followed by allergic fungal sinusitis. On the other hand, chronic sinusitis without nasal polyps is the most common in adults, followed by antrochoanal polyps. The mean duration of clinical manifestations before diagnosis in pediatric patients was significantly shorter than that in adults (P=0.001). The most common symptoms in both pediatric and adult patients were anterior nasal discharge and nasal obstruction. Proptosis was significantly higher in pediatric group than in adult group (P=0.015). On computed tomography (CT), the most commonly affected sinus in both pediatric and adult patients was the maxillary sinus followed by the anterior ethmoid sinus. Bone expansion, erosion and involvement of adjacent structures were significantly higher in pediatric patients (P=0.028, 0.027 respectively).

**Conclusion:**

Pediatric patients have a high incidence of antrochoanal polyps and allergic fungal sinusitis as unilateral inflammatory lesions. These lesions require surgical management. Inflammatory paranasal sinus lesions in pediatric patients have a shorter duration of clinical manifestations and a higher incidence of bone erosion and involvement of adjacent structures; therefore, early diagnosis and management prevent complications.

## Introduction

Pediatric unilateral chronic sinusitis has become more common among otorhinolaryngologists. Unilateral nasal manifestations should be carefully considered to reach a proper diagnosis. Various inflammatory pathologies are associated with unilateral chronic sinusitis. 

These pathologies include chronic sinusitis without nasal polyps, chronic sinusitis with nasal polyps, allergic fungal sinusitis, antrochoanal polyps, mucocele and fungal ball sinusitis. Unilateral sinonasal lesions include neoplastic lesions that are rare in children; therefore, in this study, we focused on inflammatory unilateral sinus lesions.

The aim of this study was to analyze the disease characteristics in pediatric patients with unilateral sinusitis and to compare them with those of adults. We will study demographic data, clinical manifestations and computed tomography findings.

## Materials and Methods

This prospective study was conducted at otorhinolaryngology department of our university hospital (tertiary university hospital). This study was approved by the Institutional Review Board at our university. Approval No.538-2022. All participants were informed of the study and provided written informed consent before data collection. The study included 124 patients who visited our outpatient clinic and had unilateral sinusitis between December 2022 and September 2023. The patients’ inflammatory conditions were determined based on radiological and histopathological findings. The study population was divided into pediatric (≤18 years old) and adult (>18 years old) groups. We excluded 1- patients with bilateral sinusitis 2- patients with histopathology showing invasive fungal sinusitis, benign or malignant neoplastic lesions. 3- patients with a history of sinus surgery. 4- patients with history of maxillofacial trauma.

The history was taken from all cases. The demographic characteristics of the patients were reviewed. We asked about nasal manifestations, affected side, and duration. The nasal manifestations included: nasal obstruction, anterior or postnasal discharge, headache, facial pain, allergic manifestations, smell dysfunction and epistaxis. We also asked about proptosis as an orbital manifestation. A history of dental procedures such as tooth extraction and root canal treatment was reported. Nasal endoscopic examination was performed in all cases to evaluate the presence of polyps and/or opaque and thick discharge in the nasal cavity. All patients underwent multi-slice computed tomography (CT) of the nose and paranasal sinus using a Toshiba Aquilion CT scanner. Axial and coronal 2.5 mm cuts were obtained. We assessed the affected paranasal sinuses, presence of hyperdensities within the sinus, bone expansion, bone erosion, and involvement of the adjacent structures. CT images were graded using the Lund-Mackay scoring system (1). They were graded as follows: 0=no abnormality, 1=partial opacification or 2=total opacification for each sinus. The ostiomeatal complex was scored as 0 (not occluded) or 2 (occluded). The maximum score was 12 because the patients in this study had unilateral sinusitis. 

*Statistical analysis*

SPSS 24.0 program was used for statistical analysis. The averages and standard deviations were calculated. The t- test, Mann–Whitney and Chi square tests were used for comparisons between the groups. Pearson correlation tests were used to evaluate correlations. Statistical significance was set at P ≤ 0.05.

## Results

This study included 124 patients with unilateral chronic sinusitis who were divided according to age into two groups: pediatric group ≤18 years (66 cases) and adult group >18 years (58 cases) (Table. 1).

**Table 1:** Demographic data

	**Pediatrics n=66**	**Adults n=58**	**Total n=124**	**P value**
Age (in years) Mean ± SD (Range)	13.36±3.016 (8-18)	36.31±12.728 (19-74)	24.10±14.562 (8-74)	
Males	32(48.5%)	30(51.7%)	62(50%)	0.719
Females	34(51.5%)	28(48.3%)	62(50%)	

shows the demographic data of patients in each group. In the pediatric group, the mean age of the subjects was 13.36±3.016 years (range 8-18 years). This study included 32 males and 34 females. In the adult group, the mean was 36.31±12.728 years (range 19-74 years). This study included 30 males and 28 females. There was no significant difference in sex between the groups (P=0.719). In the pediatric group, the most common inflammatory pathology was antrochoanal polyps (24 cases (36.4%)) followed by allergic fungal sinusitis (21 cases (31.8%)). There were 18 cases (27.3%) of chronic sinusitis without nasal polyps and 3 cases (4.5%) of chronic sinusitis with nasal polyps. There were no cases of mucocele or fungal balls. On the other hand, chronic sinusitis without nasal polyps was the most common inflammatory pathology in adults (18 cases (31%)) followed by antrochoanal polyps (12 cases (20.7%)). Allergic fungal sinusitis (11 cases (19%)) and chronic sinusitis with nasal polyps (10 cases (17.2%)) Figure (1). 

**Fig 1:**
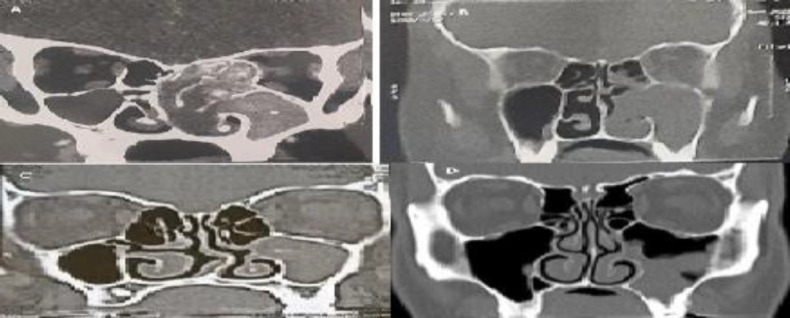
CT nose and paranasal sinuses coronal cuts of different patients with different pathologies. A: left sided allergic fungal sinusitis showing ballooning and hyperdensity areas. B: left antrochoanal polyp. C: left maxillary sinus fungal ball. D: left chronic sinusitis showing mucosal thickening

**Fig 2:**
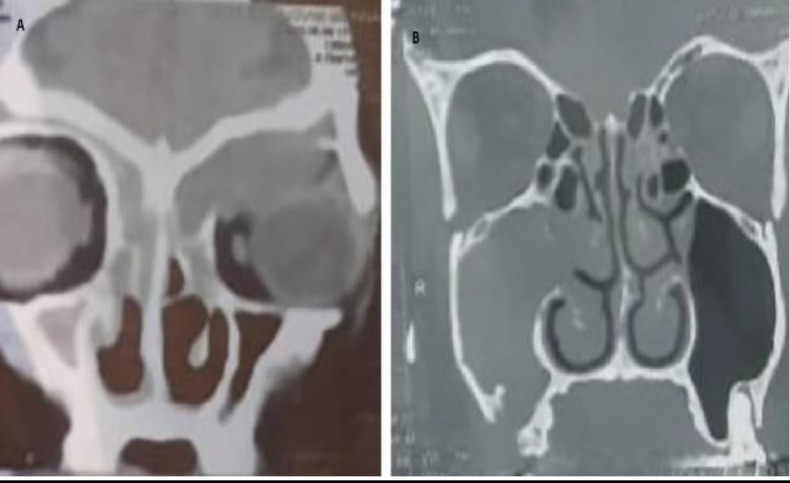
CT nose and paranasal sinuses coronal cuts of different patients with different pathologies. A: left fronto-ethmoidal mucocele pushing left eye. B: right maxillary sinusitis due to oro-antral fistula

**Fig 3:**
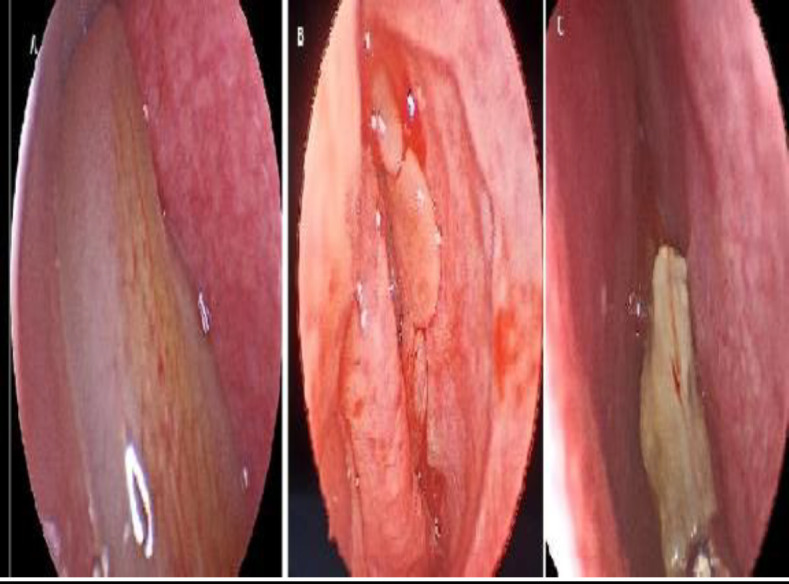
Endoscopic view of the nasal cavity. A: antrochanal polyp B: chronic sinusitis with nasal polyps. C: allergic fungal sinusitis with fungal mucin

There were 4 mucocele cases (6.9%) Figure 2 and Figure 3 cases of fungal ball sinusitis (5.2%). Table 2 shows the distribution of various pathologies among the groups.

**Table 2:** Distribution of inflammatory pathologies

**Pathology**	**Pediatrics n=66**	**Adults n=58**	**Total n=124**
Chronic sinusitis without nasal polyps	18(27.3%)	18(31%)	36(29%)
Chronic sinusitis with nasal polyps	3(4.5%)	10(17.2%)	13(10.5%)
Antrochoanal polyp	24(36.4%)	12(20.7%)	36(29%)
Mucocele	0(0%)	4(6.9%)	4(3.2%)
Allergic fungal sinusitis	21(31.8%)	11(19%)	32(25.8%)
Fungal ball sinusitis	0(0%)	3(5.2%)	3(2.4%)

The mean duration of clinical manifestations before diagnosis in pediatrics was 10.08±6.145 months, which was significantly less than the duration in adults which was 16.36±13.287 months (P=0.001). There was no significant difference in the affected side between the groups. The right: left ratio was 27:39 in the pediatric group and 27:31 in the adult group (P=0.527). As shown in Table (3), the most common symptoms in both pediatric and adult patients were anterior nasal discharge (100% in pediatric and 94.8% in adult patients) and nasal obstruction (90.9% in pediatric and 89.7% in adult patients). The next most common symptoms were posterior nasal discharge (81.8% in pediatric and 79.3% in adult patients), headache, facial pain (59.1% in pediatric and 74.1% in adult patients) and allergic manifestations (68.2% in pediatric and 69% in adult patients). The less common symptoms were smell dysfunction (18.2% in pediatric and 17.2% in adult patients) and epistaxis (18.2% in pediatric and 10.3%in adult patients). There was no significant difference in previous symptoms between pediatric and adult patients. Proptosis was significantly higher in pediatric patients (22.7%) than in adults (6.9%) P=0.015, while a history of previous dental procedures such as tooth extraction Figure (2) and root canal treatment was found in 5 cases (8.6%) in adults only. When we compared both 

groups regarding endoscopic examination Figure (3), we found no significant difference between them in findings, including nasal polyps and thick opaque nasal discharge. P=0.285, 0.793 respectively.

**Table 3:** Comparison between pediatric group and adult group regarding clinical manifestations

**Clinical manifestations**	**Pediatricsn=66**	**Adultsn=58**	**Totaln=124**	**P value**
Duration in months (mean)	10.08±6.145	16.36±13.287	13.02±10.568	0.001*
Affected side (Right: left)	27:39	27:31	54:70	0.527
Nasal obstruction	60(90.9%)	52(89.7%)	112(90.3%)	0.814
Anterior nasal discharge	66(100%)	55(94.8%)	121(97.6%)	0.061
Posterior nasal discharge	54(81.8%)	46(79.3%)	100(80.6%)	0.724
Headache and facial pain	39(59.1%)	43(74.1%)	82(66.1%)	0.077
Allergic manifestations	45(68.2%)	40(69%)	85(68.5%)	0.925
Smell dysfunction	12(18.2%)	10(17.2%)	22(17.7%)	0.891
Epistaxis	12(18.2%)	6(10.3%)	18(14.5%)	0.216
Proptosis	15(22.7%)	4(6.9%)	19(15.3%)	0.015*
Dental complaints	0(0%)	5(8.6%)	5(4%)	0.015*
Nasal polyps	48(72.7%)	37(63.8%)	85(68.5%)	0.285
Thick & opaque discharge	36(54.5%)	33(56.9%)	69(55.6%)	0.793

**Fig 4:**
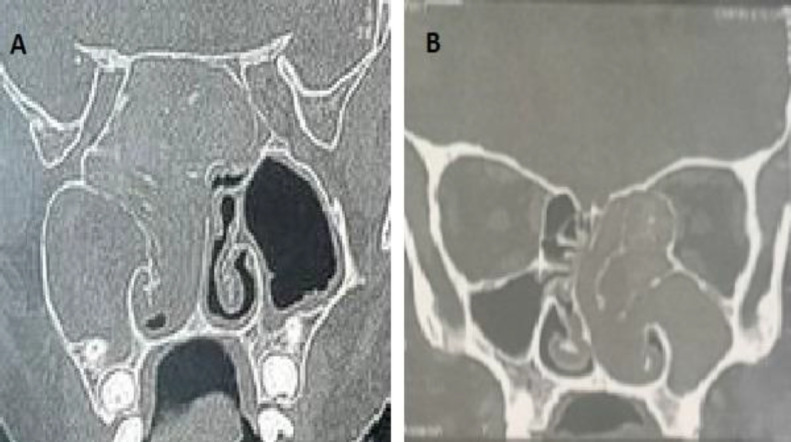
CT nose and paranasal sinuses coronal cuts showing ballooning of paranasal sinuses in allergic fungal sinusitis A: right allergic fungal sinusitis (pediatric). B: left allergic fungal sinusitis (adult)

**Fig 5:**
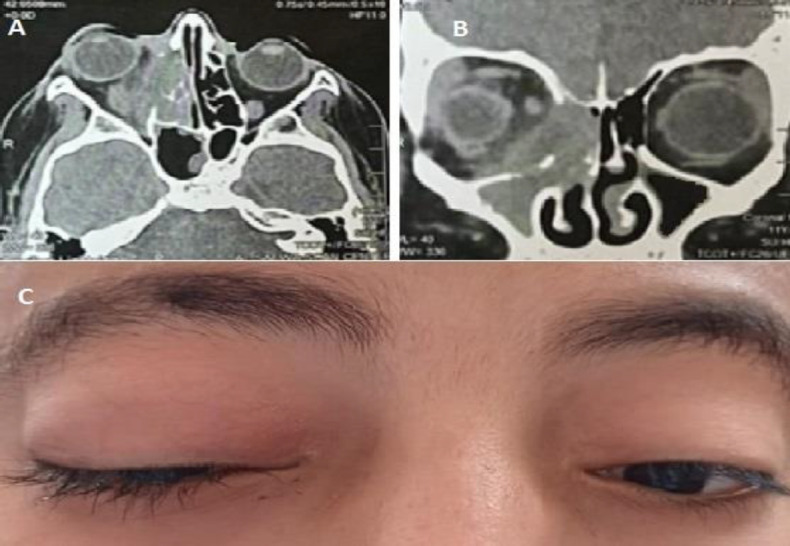
Right allergic fungal sinusitis with bone erosion and intra orbital extension causing proptosis. A: CT nose axial cut B: CT nose coronal cut C: anterior view of patient showing right proptosis

On computed tomography (CT), the most common affected sinus in both pediatric and adult patients was the maxillary sinus (90.9% in pediatric patients and 82.8% in adults) followed by the anterior ethmoid sinus (81.8% in pediatric patients and 81% in adults). Posterior ethmoid sinus (72.7% in pediatrics and 67.2% in adults) and sphenoid sinus (54.5% in pediatrics and 60.3% in adults). Lastly, the frontal sinus was affected (31.8% in pediatric patients and 22.4% in adults). There was no significant difference between the two groups regarding the affected paranasal sinuses as shown in Table (4). There was no significant difference in the presence of calcifications and radiopaque shadows between the two groups (P= 0.343). Bone expansion, bone erosion, and involvement of adjacent structures were significantly higher in pediatric group (P=0.028, 0.027 respectively). Bone expansion and erosion occurred in patients with allergic fungal sinusitis patients. Figures (4,5). The mean Lund-Mackay score was 7.97±2.914 in pediatrics and 6.19±2.982 in adults. This difference was statistically significant (P=0.001).

**Table 4:** Comparison between pediatric group and adult group regarding CT findings

**CT findings**	**Pediatrics n=66**	**Adults n=58**	**Total n=124**	**P value**
Anterior ethmoid sinus opacity	54(81.8%)	47(81%)	101(81.5%)	0.911
Posterior ethmoid sinus opacity	48(72.7%)	39(67.2%)	87(70.2%)	0.505
Maxillary sinus opacity	60(90.9%)	48(82.8%)	108(87.1%)	0.177
Sphenoid sinus opacity	36(54.5%)	35(60.3%)	71(57.3%)	0.515
Frontal sinus opacity	21(31.8%)	13(22.4%)	34(27.4%)	0.241
Hyperdensities within the sinus	21(31.8%)	14(24.1%)	35(28.2%)	0.343
Bone expansion & thinning	20(30.3%)	8(13.8%)	28(22.6%)	0.028*
Bone erosion & involvement of adjacent structures	12(18.2%)	3(5.2%)	15(12.1%)	0.027*
Lund-Mackay score (mean)	7.97±2.914	6.19±2.982	7.14±3.067	0.001*

## Discussion

Unilateral sinonasal lesions are not uncommon. These lesions can be classified into two major categories: inflammatory and neoplastic. Since this study mainly focuses on pediatric patients and compares them to adults, we will include inflammatory pathologies only because neoplastic lesions are rare in pediatrics. The mean age of subjects is 13.36±3.016 years in the pediatric group and 36.31±12.728 years in adults. There is no significant difference in gender between both groups (P=0.719). Some studies found that unilateral sinonasal diseases were more common in males (2). While others found that there was no gender difference (3). There are various pathologies of unilateral chronic sinusitis including chronic sinusitis without nasal polyps, chronic sinusitis with nasal polyps, allergic fungal sinusitis, antrochoanal polyps, mucocele and fungal ball sinusitis. In the pediatric group, the most common unilateral inflammatory pathology was antrochoanal polyps (36.4%) followed by allergic fungal sinusitis (31.8%) then chronic sinusitis without nasal polyps (27.3%). In adults group, chronic sinusitis without nasal polyps was the most common (31%) followed by antrochoanal polyps (20.7%) then allergic fungal sinusitis (19%). In a study by Satish Nair et al., the most common unilateral inflammatory lesions were chronic sinusitis (43.2%) followed by antrochoanal polyps (20.5%) then allergic fungal sinusitis (15.9%) (4). Many studies have stated that chronic rhinosinusitis is the most common unilateral inflammatory sinonasal disease (5-8). McClay JE et al. stated that allergic fungal sinusitis occurred unilaterally in 70% of pediatric patients, while it occurred unilaterally in 37% of adults (9). Dong Hoon Lee et al. stated that the incidence of antrochoanal polyps was nearly equal in pediatrics (48.2%) and in adults (51.8%) (10). In our study, there were no mucocele or fungal ball cases in pediatrics. Mucoceles occurred mainly in adults (mean age of 52.7 years) in a study done by Ta- Jen Lee et al. (11). Fungal balls were more common in adults. Unilateral maxillary sinus fungal ball occurs in immunocompetent middle aged or elderly patients and rarely in children (12).

The mean duration of clinical manifestations before diagnosis in pediatric patients was 10.08±6.145 months, while in adults it was 16.36±13.287 months, so it was significantly shorter in pediatric patients than in adults (P=0.001). Some studies found that the duration of symptoms before diagnosis was 11 months in allergic fungal sinusitis (13). The lesser duration in pediatrics is likely due to the smaller size of paranasal sinuses and nasal cavity. Also the weakness of the bone plays a role in the early development of complications. There was no significant difference in the affected side between both groups in our research. The right: left ratio was 27:39 in the pediatric group and 27:31 in the adult group (P=0.527). The right side was predominant in a study done by Azzam (2). In this study, the most common symptoms in both pediatrics and adults were anterior nasal discharge (100% in pediatrics and 94.8% in adults) and nasal obstruction (90.9% in pediatrics and 89.7% in adults). The next common symptoms were posterior nasal discharge, headache, facial pain and allergic manifestations. The less common symptoms were smell dysfunction and epistaxis. There was no significant difference in the clinical symptoms between pediatrics and adults. In a study done by Satish et al., 87% of patients presented with nasal obstruction, 72% of patients presented with anterior nasal discharge and 57% of patients with postnasal discharge. Smell dysfunction was in 19% of cases and epistaxis was in 17% of cases. Satish et al found that in unilateral inflammatory conditions, nasal obstruction and nasal discharge are more common presentations than epistaxis and orbital manifestations such as proptosis. On the other hand, epistaxis and orbital manifestations such as proptosis and limited extra-ocular motility are more common in neoplastic conditions (4). In our study, proptosis was significantly higher in pediatrics (22.7%) than in adults (6.9%) with P=0.015. As we saw in the literature, in 42% of pediatrics with allergic fungal sinusitis, there was altered facial skeleton (proptosis, telecanthus, or malar flattening) compared to 10% for adults. This is explained by the more pliability of bony structure in pediatrics (9,13,14). 

Mucocele commonly presents with orbital manifestations including ptosis and proptosis (11). Smell dysfunction is uncommon as the diseases are unilateral. In this study, dental complaints were found in 5 cases (8.6%) in the adults group only. A history of dental complaints or previous dental procedures occurs mainly in adults. It is usually associated with maxillary sinusitis. Oroantral fistula after tooth extraction is a cause. Some researches stated that when odontogenic treatment penetrates the floor of the maxillary sinus, this increases the risk of maxillary sinus fungal ball. During root canal treatment, the filling agent contains zinc oxide which promotes the growth of fungi (15, 16). When we compared both groups regarding endoscopic examination, we found no significant difference between them in findings including nasal polyps and thick opaque nasal discharge. P=0.285, 0.793 respectively. The presence of multiple polyps usually indicates ethmoidal polyps. Antrochoanal polyp presents as a solitary polyp originating from the maxillary antrum passing into the nasal cavity then towards the choana (17). Cases of fungal sinusitis are characterized by the presence of thick opaque discharge (fungal mucin)(18). 

In computed tomography (CT), the most common affected sinus in both pediatrics and adults was the maxillary sinus (90.9% in pediatrics and 82.8% in adults) followed by the anterior ethmoid sinus (81.8% in pediatrics and 81% in adults). Then, the posterior ethmoid sinus, sphenoid sinus and the frontal sinus respectively. There was no significant difference between both groups regarding the affected paranasal sinus in this study. In a study done by Chung et al, unilateral sinonasal lesions involved the maxillary sinus in 96% of cases and the anterior ethmoid in 69% of cases (3).

In our study, 31.8% of cases showed hyperdensities and calcifications inside the paranasal sinus in the pediatric group while 24.1% of cases in the adult group (P=0.343). 

In pediatrics group, 30.3% of cases showed bone expansion while in adults group there were 13.8% of cases (P=0.028). The presence of hyperdensities within the sinus and bone expansion was more common in cases of allergic fungal sinusitis than other types of chronic sinusitis. In cases of fungal balls, there was a characteristic CT finding in the form of sinus hyperdensity or calcification (19). 18.2% of cases showed bone erosion and involvement of adjacent structures in the pediatric group, a significantly higher percentage than in adults (5.2% of cases) (P=0.027). Bone expansion, erosion and extension to surrounding structures occurred in cases of allergic fungal sinusitis. Ghegan et al stated that 56% of cases of allergic fungal sinusitis showed bone expansion and/or erosion and intra-orbital extension while it occurred in only 5% of other forms of chronic rhinosinusitis (20). 

McClay JE et al, found that pediatric patients had a higher incidence of bone expansion, erosion, intra-orbital and intracranial extension in allergic fungal sinusitis. The reason for that is the thinner bone in pediatrics than in adults (9). Mucoceles have the CT appearance of full sinus opacification with bone expansion either rounded or ovoid and/or bone erosion. The bone remodels due to increased pressure within the sinus cavity (21). 

Inflammatory conditions like allergic fungal sinusitis, and mucocele can present with bone erosion and involvement of surrounding structures giving a picture resembling neoplastic conditions (4). In our study, the Lund-Mackay score mean was 7.97±2.914 in pediatrics and 6.19±2.982 in adults. That was statistically significant (P=0.001). This is due to the smaller size of paranasal sinuses in pediatrics so they are easily opacified either due to secretions or polyps. This more or less agreed with the study done by Chung et al, where the mean Mackay score was 6.2 ± 2 (3). 

## Conclusion

Patients with unilateral nasal manifestations should be taken with great care especially pediatrics. In our tertiary hospital experience, pediatric patients have a high incidence of antrochoanal polyps and allergic fungal sinusitis as unilateral inflammatory paranasal sinus lesions. These lesions require surgical management. Unilateral inflammatory paranasal sinus lesions in pediatrics have a shorter duration of clinical manifestations and a higher incidence of complications as in allergic fungal sinusitis due to bone erosion and involvement of adjacent structures, so early diagnosis and management prevent possible complications.
